# Comparative Analysis of Deep Learning Approaches for Histopathology-Based Survival Prediction in Hepatocellular Carcinoma

**DOI:** 10.3390/cancers18101534

**Published:** 2026-05-09

**Authors:** Sung Hak Lee, Kwangil Yim, Hyun-Jong Jang

**Affiliations:** 1 Department of Hospital Pathology, Seoul St. Mary’s Hospital, College of Medicine, The Catholic University of Korea, Seoul 06591, Republic of Korea; hakjjang@catholic.ac.kr; 2 Department of Hospital Pathology, Uijeongbu St. Mary’s Hospital, College of Medicine, The Catholic University of Korea, Uijeongbu 11765, Republic of Korea; kangse_manse@catholic.ac.kr; 3 Department of Physiology, College of Medicine, The Catholic University of Korea, Seoul 06591, Republic of Korea

**Keywords:** deep learning, foundation model, hepatocellular carcinoma, overall survival, pathology, prognosis

## Abstract

Background: Accurate prognostic stratification in hepatocellular carcinoma (HCC) remains challenging due to substantial tumor heterogeneity. Deep learning (DL)-based approaches, particularly convolutional neural networks (CNNs), have been widely applied for prognostic prediction using histopathological images. Recently, pathology foundation models combined with multiple-instance learning (MIL) have emerged as a promising alternative. In this study, we aimed to provide a comparative evaluation of conventional CNN-based and foundation model-based approaches for HCC prognosis prediction, with a focus on tissue selection strategies and cross-dataset generalizability. Methods: We compared a patch-level CNN approach with a foundation model-based MIL approach for overall survival (OS) prediction from hematoxylin and eosin-stained whole-slide images. A total of 256 patients from Seoul St. Mary’s Hospital (SSMH) and 334 patients from the TCGA dataset were included. Models were trained using either all tissue regions or tumor-only regions under five-fold cross-validation. Model performance was evaluated using the concordance index (C-index), Kaplan–Meier analysis, and time-dependent receiver operating characteristic analysis. Cross-dataset validation and combined-dataset training assessed generalizability. Results: In the SSMH dataset, the CNN model performed better with tumor-only regions (C-index 0.8308) than with all tissue regions (0.7498). In contrast, the foundation model-based MIL approach showed stable performance regardless of input regions (C-indices: 0.8701 and 0.8752). Similar stability was observed in the TCGA dataset (0.7744 and 0.7722). Cross-dataset validation showed reduced performance, indicating limited generalizability. Combining datasets did not lead to performance improvement. Subgroup analyses showed prognostic information beyond histologic grade, and feature visualization revealed relevant histopathologic patterns. Conclusions: A foundation model-based MIL approach provides robust and interpretable prognostic modeling in HCC. DL-based prediction offers complementary information beyond conventional clinicopathological variables. Future efforts integrating multimodal data and improving generalizability will be essential for clinical translation.

## Simple Summary

Accurate outcome prediction in hepatocellular carcinoma remains challenging due to tumor heterogeneity. We compared two deep learning approaches based on histopathological whole-slide images: a conventional patch-based model and a foundation model with multiple-instance learning. The foundation model demonstrated stable performance across different tissue settings, whereas the conventional model performed poorly when all tissue regions were used. Performance decreased across datasets, highlighting challenges associated with data heterogeneity. Importantly, the model captured prognostic information beyond tumor grade and identified meaningful histopathologic patterns. These findings suggest that foundation model-based approaches provide robust and interpretable prognostic information and may complement clinical assessment.

## 1. Introduction

Hepatocellular carcinoma (HCC) is one of the most prevalent and lethal malignancies worldwide, ranking as the third leading cause of cancer-related death globally and representing the most common primary liver malignancy [1]. The incidence of HCC continues to rise in many regions, driven by chronic liver diseases such as viral hepatitis, alcohol-related liver disease, and non-alcoholic fatty liver disease [2]. Despite advances in surveillance and therapeutic strategies, the prognosis of HCC remains poor, largely due to late-stage diagnosis, high recurrence rates, and heterogeneous tumor biology [3]. Therefore, accurate prognostic stratification is essential for optimizing treatment decisions, guiding surveillance strategies, and identifying patients who may benefit from adjuvant therapies. However, reliable prognostic stratification in HCC remains challenging due to its marked heterogeneity at the histological, molecular, and genetic levels [4,5].

Histopathological evaluation has long been a cornerstone of prognostic assessment in oncology. Tumor differentiation, architectural patterns, and microenvironmental features reflect the biological behavior of various cancers and are closely associated with clinical outcomes [6,7,8]. Both classical pathological features—such as tumor grade, microvascular invasion, and cellular atypia—and emerging histomorphological characteristics, including macrotrabecular patterns, tertiary lymphoid structures, and vessels encapsulating tumor clusters, have demonstrated significant associations with survival outcomes in HCC [9,10,11]. These findings suggest that hematoxylin and eosin (H&E)-stained whole-slide images (WSIs) contain rich phenotypic information that reflects the underlying molecular and cellular processes of tumor progression [12]. However, many of these features remain difficult to quantify reproducibly using conventional pathology workflows, highlighting the need for computational approaches to extract clinically meaningful information from histological images.

With the advent of digital pathology and deep learning (DL), there has been increasing interest in leveraging WSIs for automated prognostic modeling. Convolutional neural networks (CNNs) have traditionally been used to extract complex features from digitized WSIs and have been applied to a variety of histopathological image analysis tasks, including tumor classification, mutation prediction, and survival estimation [13]. In HCC, CNN-based approaches have been widely used to predict recurrence and survival across multiple studies [14,15,16,17,18,19]. Nevertheless, conventional CNN-based approaches are often limited by their reliance on large annotated datasets and their limited ability to generalize across diverse cohorts.

Recently, pathology foundation models trained on large-scale histopathological datasets have emerged as a promising alternative to traditional CNN-based methods. Unlike CNNs, these foundation models are pre-trained on massive and diverse datasets to learn generalizable representations of tissue morphology, enabling improved performance across a variety of downstream tasks with limited supervision [20,21]. In particular, foundation models such as UNI2 provide high-quality patch-level embeddings that capture both cellular and architectural features, which are critical for prognostic assessment [22]. When combined with multiple-instance learning (MIL) frameworks such as clustering-constrained attention multiple-instance learning (CLAM), these embeddings can be effectively aggregated into slide-level representations for various digital pathology tasks [23]. Compared with conventional CNN-based pipelines, this approach has the potential to better capture complex spatial patterns and the hierarchical organization of tumor tissues, thereby improving prognostic modeling.

Given these advances, a systematic comparison between a conventional CNN-based approach and a foundation model-based MIL approach in the context of HCC prognosis prediction is warranted. In this study, we aimed to (1) compare the prognostic performance of an Inception v3-based CNN model and a foundation model-based pipeline utilizing UNI2 embeddings combined with CLAM-based MIL, (2) evaluate the impact of tissue selection strategies (all-tissue vs. tumor-only), and (3) assess model robustness and generalizability across independent datasets. Through this analysis, we sought to provide practical insights into the strengths and limitations of current DL paradigms for HCC prognosis prediction. In addition, we investigated prognostically relevant pathologic features of HCC using visualization methods applied to both CNN and UNI2 embeddings.

## 2. Materials and Methods

H&E-stained slides from patients who underwent HCC resection at Seoul St. Mary’s Hospital were collected for this study, hereafter referred to as the SSMH dataset. Slides with a tumor area smaller than 3.24 mm^2^ were excluded from further analysis. Tumor area was estimated using a CNN model developed in our previous study to distinguish between normal and tumor tissue patches [24]. The defined tumor area corresponds to 100 tissue image patches of 360 × 360 pixels at 20× magnification. A total of 256 patients were included in this study, with a median age at diagnosis of 61 years (range, 35–85). Among these patients, 34 patients died, whereas the remaining 222 patients were censored. The median survival time was 19 months (range, 1–119) in deceased patients and 21 months (range, 1–208) in censored patients.

To compare the performance of DL models across different datasets, HCC slides from the TCGA-LIHC project were downloaded from the National Cancer Institute Genomic Data Commons Data Portal (https://portal.gdc.cancer.gov/) and are hereafter referred to as the TCGA dataset. Slides with a tumor area of less than 3.24 mm^2^ were also excluded. In total, 334 patients were included, with a median age at diagnosis of 61 years (range, 16–90). In the TCGA dataset, 123 patients died, with a median survival time of 14 months (range, 1–107), whereas the remaining 211 patients were censored, with a median survival time of 22 months (range, 1–120).

**Figure 1:**
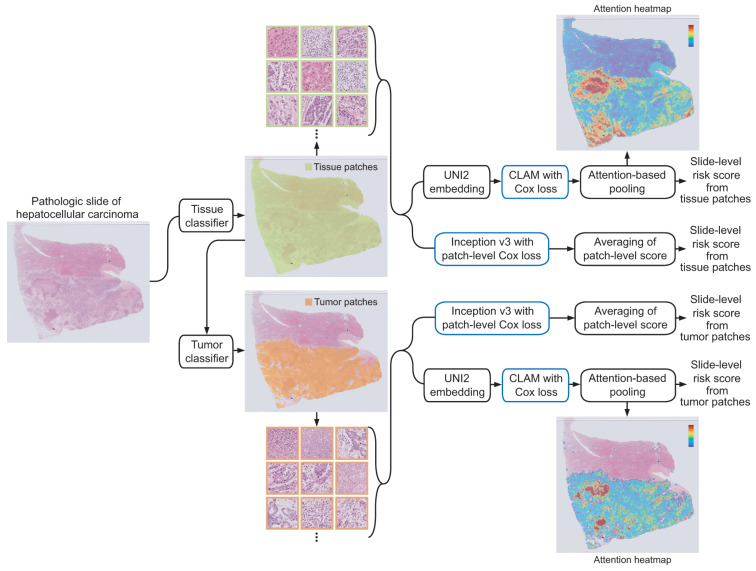
Experimental procedure. Whole-slide images are divided into 360 × 360 pixel image patches at 20× magnification. In the upper pathway, all tissue patches are used for prognosis prediction, whereas in the lower pathway, only tumor patches are used. The blue box indicates where model training occurs.

We selected Inception v3 and CLAM with UNI2 embeddings as representative models for CNN-based and foundation model-based MIL approaches, respectively, because they demonstrated the best performance in our experimental design (Supplementary Table S1). First, an Inception v3 model was trained to predict risk scores based on overall survival (OS) using 360 × 360 pixel tissue image patches at 20× magnification. In this patch-based approach, the network was trained to predict a continuous risk score for each image patch within a WSI. All patches extracted from the same WSI were assigned the corresponding slide-level survival time and event (death) indicator. The model parameters were optimized by minimizing the negative partial log-likelihood of the Cox proportional hazards model, computed at the patch level. The final slide-level risk score was obtained by averaging the risk scores across all patches within the WSI. Unlike attention-based MIL models that explicitly aggregate patch-level features into slide-level representations, this patch-level approach directly optimizes the survival objective at the instance level, implicitly assuming equal contribution of all patches. Although patches from the same slide are not statistically independent, this formulation has been widely adopted as a practical approximation for slide-level labels. In WSIs, various non-tissue artifacts—including air bubbles, compression artifacts, out-of-focus blur, pen markings, tissue folding, and background regions—can hinder the effective learning of prognostic features. To address this issue, we applied a CNN model to identify and exclude non-tissue image patches, retaining only valid tissue regions for analysis (Figure 1, upper pathway). The CNN was composed of three convolutional-pooling layers and was trained to classify tissue versus non-tissue patches. The convolutional layers consisted of 12, 24, and 24 filters (each with a kernel size of 5 × 5), each followed by a 2 × 2 max-pooling layer. For training, 10,000 improper image patches containing various artifacts and 10,000 proper tissue patches were manually selected. Furthermore, only tumor patches were selected to investigate whether prognosis-relevant features are primarily located in tumor regions rather than in normal tissue (Figure 1, lower pathway).

Accordingly, models were trained using either all tissue patches or tumor-only patches under a five-fold cross-validation scheme. During training, data augmentation techniques were applied to the tissue image patches, including random 90° rotations, random horizontal and vertical flipping, and random color jittering (with variations of less than 10% in each color channel). From these augmented image patches, patch-level features were extracted by Inception v3, a convolutional neural network architecture designed to efficiently capture multi-scale visual patterns. Inception v3 consists of stacked Inception modules that perform parallel convolutions with multiple kernel sizes (e.g., 1 × 1, 3 × 3, and factorized convolutions), allowing the network to capture both fine-grained cellular details and broader tissue architecture within histopathological images.

For attention-based MIL survival modeling, specifically using the CLAM framework, each WSI was represented as a bag of patch-level feature embeddings extracted using a pretrained UNI2 encoder [22,23]. Before embedding, stain normalization was performed on all tissue image patches using the Vahadane method to reduce inter-slide variability in staining [25]. In preliminary experiments conducted prior to the main study, we confirmed that stain normalization was essential for achieving stable performance of the UNI2/CLAM model. In contrast to the training of the Inception v3 model, data augmentation techniques such as random color jittering were not applied in this setting, because embedding was completed prior to the training step. An attention-based MIL pooling mechanism was employed to aggregate patch-level features into a slide-level representation. Specifically, for a slide i with a set of patch embeddings $B_{i}=\{x_{i1},\ldots ,x_{iN_{i}}\}$, the attention weight $a_{ik}$ for each patch was computed and normalized using a softmax function, and the slide-level representation was obtained as a weighted sum of patch features:

$$z_{i}=\sum_{k=1}^{N_{i}}a_{ik}\;\phi (x_{ik}),$$

where ϕ(⋅) denotes a learnable feature transformation. The aggregated representation $z_{i}$ was then mapped to a scalar risk score $r_{i}=f_{\theta }(z_{i})$, with higher values indicating increased hazard. Model parameters were optimized by minimizing the negative partial log-likelihood of the Cox proportional hazards model. In summary, the model jointly learns attention-based slide representations and prognostic risk scores in an end-to-end manner using the Cox proportional hazards objective. When the trained model is used for survival prediction, the slide-level risk score can be obtained by applying attention-based MIL pooling to aggregate patch features into a slide representation, followed by a Cox proportional hazards layer to estimate the risk. The MIL models were also trained using either all tissue patches or tumor-only patches in WSIs. Detailed information on model training and hyperparameter settings is summarized in Supplementary Table S2.

Because models were trained independently in each cross-validation fold, the predicted risk scores were not directly comparable across folds due to differences in model calibration and scaling. Although the underlying survival distributions across folds were similar, patients with comparable survival outcomes could receive substantially different risk scores depending on the fold-specific model. To address this issue, fold-wise standardization was applied to align the scale of predictions while preserving the relative ranking within each fold. This enabled the aggregation of predictions into a unified dataset for downstream survival analyses. Specifically, within each validation fold, risk scores were normalized using a z-score transformation based on the mean and standard deviation of that fold:

$$z_{i}=\frac{r_{i}-\mu_{f}}{\sigma_{f}}$$

where $r_{i}$ denotes the predicted risk score for sample i, and $\mu_{f}$ and $\sigma_{f}$ are the mean and standard deviation of risk scores within fold f.

The standardized scores from all folds were subsequently combined into a unified dataset for downstream survival analyses, including Kaplan–Meier (KM) estimation, Cox proportional hazards modeling, and time-dependent receiver operating characteristic (ROC) analysis. This approach preserved the relative ranking of patients within each fold while enabling comparability across folds. Consequently, the validation results from the five folds were integrated into a single unified validation set encompassing all samples in the dataset. Overall model performance was evaluated using Harrell’s concordance index (C-index), which measures the agreement between predicted risk scores and observed survival outcomes. The C-index was calculated as the proportion of all comparable patient pairs in which the predicted risk scores were correctly ordered with respect to survival times, accounting for censoring.

**Figure 2:**
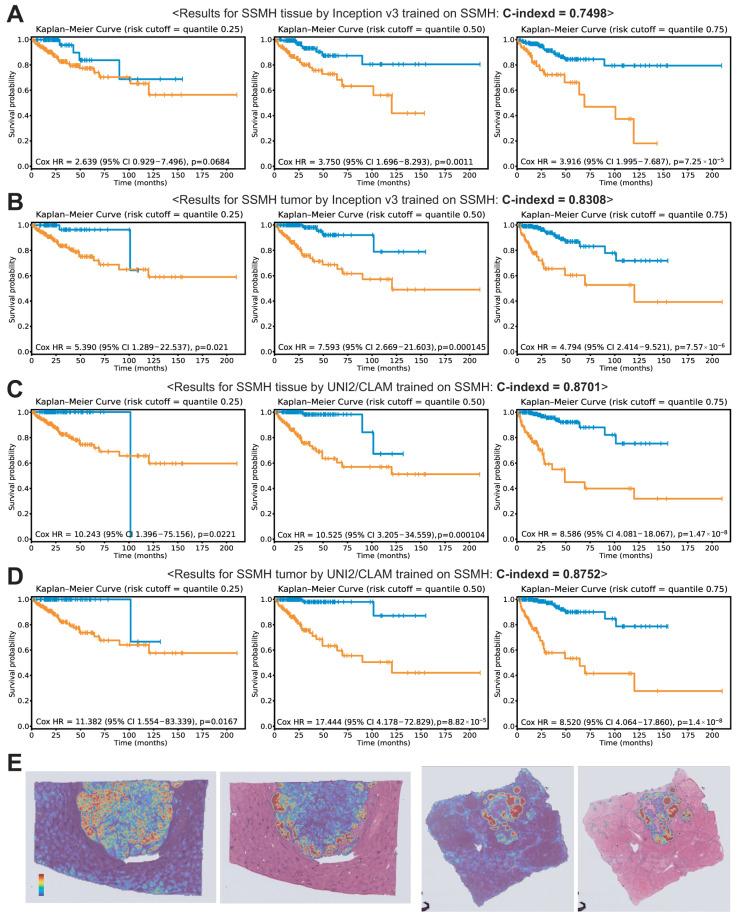
Results for the SSMH dataset. Kaplan–Meier (KM) curves are shown for risk groups stratified by the 25th, 50th, and 75th percentiles of the model-predicted risk scores: (**A**) KM curves for Inception v3 trained on all tissue patches. (**B**) KM curves for Inception v3 trained on tumor patches. (**C**) KM curves for UNI2/CLAM trained on all tissue patches. (**D**) KM curves for UNI2/CLAM trained on tumor patches. (**E**) Attention heatmaps for cases with good (**left**) and poor (**right**) prognosis.

To ensure robustness and minimize bias associated with data-driven cutoff selection, patients were stratified using predefined quantile-based thresholds (25th, 50th, and 75th percentiles of the risk score) into low- and high-risk groups (Figure 2). KM survival curves were constructed for each cutoff to compare survival differences between risk groups.

To evaluate the discriminatory performance of the survival model at clinically relevant time points, we performed time-dependent ROC analysis at 1-, 3-, and 5-year horizons. Event status (death vs. censoring) was used to construct structured survival data. Time-dependent area under the curve (AUC) values were computed using the cumulative/dynamic AUC method with inverse probability of censoring weighting, as implemented in the scikit-survival framework.

Visualization methods such as t-distributed stochastic neighbor embedding (t-SNE) have been widely used to investigate task-specific feature representations captured by DL models. We applied t-SNE to features from the last fully connected layer of Inception v3 (2048 dimensions) and to UNI2 embeddings (1536 dimensions) to reveal prognosis-related histopathologic characteristics of HCC. For both approaches, 25 low-risk and 25 high-risk cases that were accurately predicted by the DL models were selected for t-SNE visualization. For Inception v3, 200 patches per slide, whose risk scores were well aligned with their corresponding prognostic labels, were used. For UNI2, patches within the top 10% of attention scores were selected for t-SNE visualization. When the number of selected patches exceeded 200, only the top 200 patches with the highest attention scores were used.

The difference in C-index between models was assessed using bootstrap resampling (1000 iterations). For each iteration, patients were sampled with replacement, and the ΔC-index was calculated. The 95% confidence interval and *p*-value were derived from the bootstrap distribution.

All DL models were implemented using PyTorch (version 2.4.1) in Python (version 3.10; Python Software Foundation, Wilmington, DE, USA). Survival analyses, including Kaplan–Meier estimation and time-dependent ROC analysis, were performed using the scikit-survival library (version 0.25.0). Model training was conducted on three computers equipped with NVIDIA GeForce RTX 3090 GPUs (24 GB VRAM; NVIDIA Corporation, Santa Clara, CA, USA).

## 3. Results

We aimed to determine whether integrating a pathology foundation model (UNI2) with a CLAM framework provides superior predictive power compared to traditional CNN-based feature extraction for HCC prognostic stratification. A secondary objective was to evaluate how the inclusion of diverse tissue components, as opposed to isolated tumor regions, affects the robustness and discriminative power of these prognostic models (Figure 1). These objectives were first evaluated using our internal dataset (SSMH dataset).

When Inception v3 was trained on all tissue image patches to predict prognosis in the SSMH dataset, the C-index was 0.7498. KM curves were constructed based on the 25th, 50th, and 75th percentiles of the model-predicted risk scores, and the corresponding Cox proportional hazards ratios (HRs) were 2.639, 3.750, and 3.916 for the 25th, 50th, and 75th percentiles, respectively (Figure 2A). Performance improved when training and validation were restricted to tumor patches, with a C-index of 0.8308 (*p* < 0.05). The corresponding HRs were 5.390, 7.593, and 4.794 for the 25th, 50th, and 75th percentiles, respectively (Figure 2B).

When UNI2 embeddings were used within the CLAM framework, the C-indices were 0.8701 and 0.8752 for the all-tissue and tumor-only paradigms, respectively (Figure 2C,D). In contrast to the Inception v3 models, no significant performance difference was observed between the all-tissue and tumor-only paradigms. Although the performance of the two models was not statistically superior to that of the Inception v3 model trained on tumor tissues (both *p* ≈ 0.15), these findings suggest that the UNI2 embeddings within the CLAM framework provide stable predictive performance, regardless of whether all-tissue or tumor-only paradigms are used. The corresponding HRs for the 25th, 50th, and 75th percentiles were 10.243, 10.525, and 8.586 for the all-tissue paradigm, and 11.382, 17.444, and 8.520 for the tumor-only paradigm, respectively. Representative attention heatmaps for cases with good (left) and poor (right) prognosis are shown in Figure 2E. In each case, the left image corresponds to the all-tissue paradigm, whereas the right image corresponds to the tumor-only paradigm.

**Figure 3:**
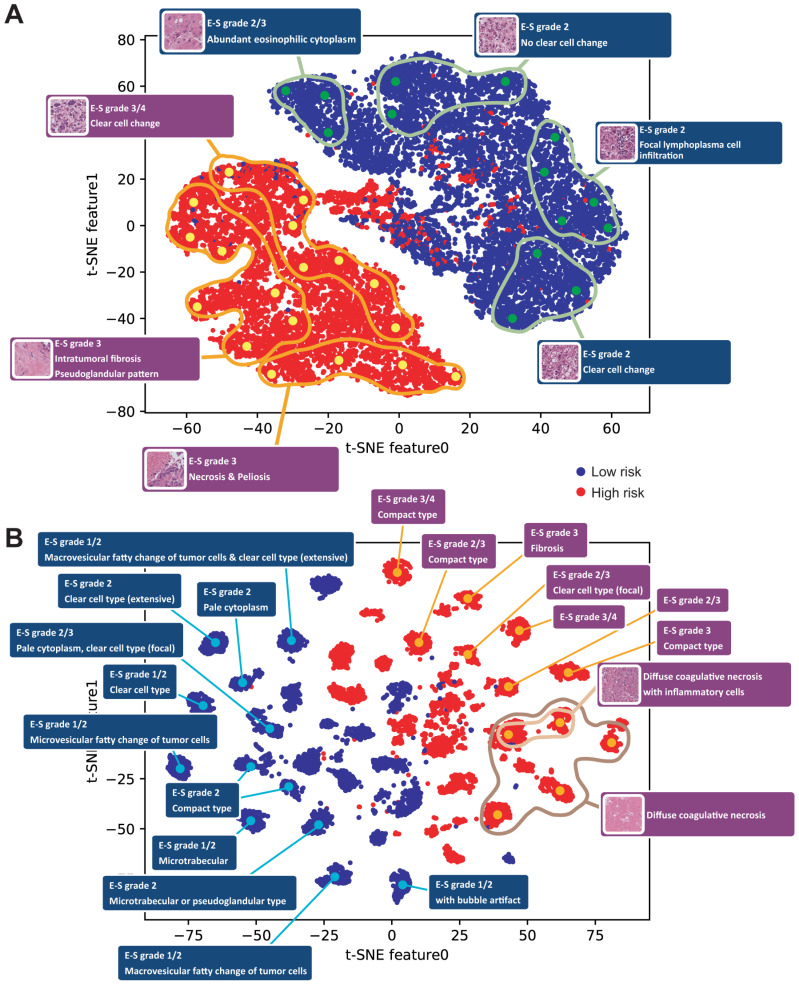
Visualization of patch embeddings from low- and high-risk patients using t-distributed stochastic neighbor embedding (t-SNE). (**A**) t-SNE plot of Inception v3 features. (**B**) t-SNE plot of UNI2 embeddings.

We applied t-SNE to investigate prognosis-related histopathologic features (Figure 3). Because the Inception v3 model performed poorly under the all-tissue paradigm, this analysis was restricted to models trained using the tumor-only paradigm. For both Inception v3 features and UNI2 embeddings, separation between patches from low- and high-risk cases was observed. However, notable differences were identified between the two representations. In the case of Inception v3 features, data points were diffusely distributed across the plot (Figure 3A), whereas for UNI2 embeddings, points were clustered into clearly separable groups (Figure 3B). To gain further insights, we analyzed histopathologic features at selected regions in the plots (highlighted by large dots). We defined specific coordinates and extracted image patches located around these positions. These patches were reviewed by pathologists (K.Y. and S.H.L.) to assess their histopathologic characteristics. Due to the diffuse distribution of Inception v3 features, distinct characteristics could not be clearly distinguished at individual points. Therefore, neighboring points were grouped into small clusters to describe representative features (Figure 3A). In contrast, for UNI2 embeddings, distinct histopathologic characteristics could be clearly identified at specific points (Figure 3B). Common high-risk features observed in both plots included high histologic grade, fibrosis, and necrosis. The UNI2 embedding plot revealed distinct features associated with low risk, including fatty change in tumor cells and a microtrabecular pattern. Although the original distribution of grade 1/2 and 3/4 tumors was 36% and 64%, respectively, 91% of low-risk patches were grade 1/2, whereas 86% of high-risk patches were grade 3/4. Because only three image patches contained tertiary lymphoid structures, the model may not have learned to recognize them as important determinants of survival. Necrosis was observed in 7% of poor-prognosis patches but in only 1% of good-prognosis patches.

**Figure 4:**
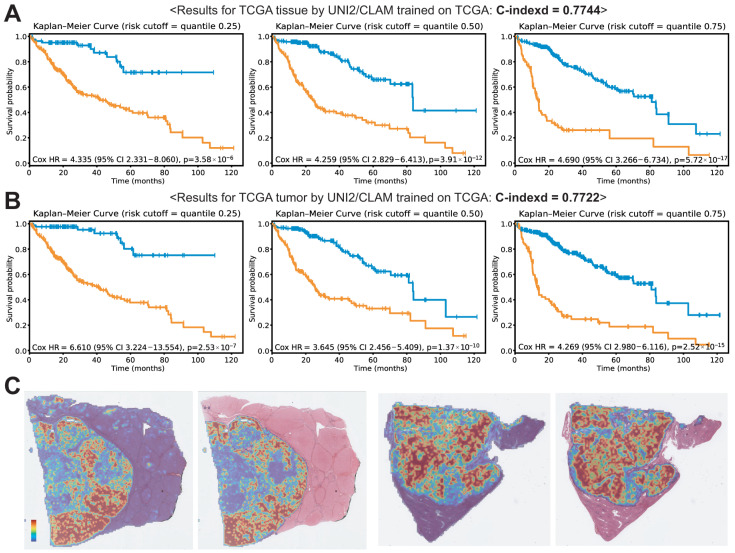
Results for the TCGA dataset. Kaplan–Meier (KM) curves are shown for risk groups stratified by the 25th, 50th, and 75th percentiles of the model-predicted risk scores. (**A**) KM curves for UNI2/CLAM trained on all tissue patches. (**B**) KM curves for UNI2/CLAM trained on tumor patches. (**C**) Attention heatmaps for cases with good (**left**) and poor (**right**) prognosis.

Next, we trained prognostic models on the TCGA dataset using the UNI2/CLAM framework to determine whether this approach is applicable to prognostic prediction in other datasets. The C-indices were 0.7744 and 0.7722 for the all-tissue and tumor-only paradigms, respectively (Figure 4A,B). Consistent with the models trained on the SSMH dataset, no significant difference was observed between UNI2/CLAM models trained using the all-tissue and tumor-only paradigms. The corresponding HRs for the 25th, 50th, and 75th percentiles were 4.335, 4.259, and 4.690 for the all-tissue paradigm, and 6.610, 3.645, and 4.269 for the tumor-only paradigm, respectively. Representative attention heatmaps for cases with good (left) and poor (right) prognosis in the TCGA dataset are also provided in Figure 4C.

**Figure 5:**
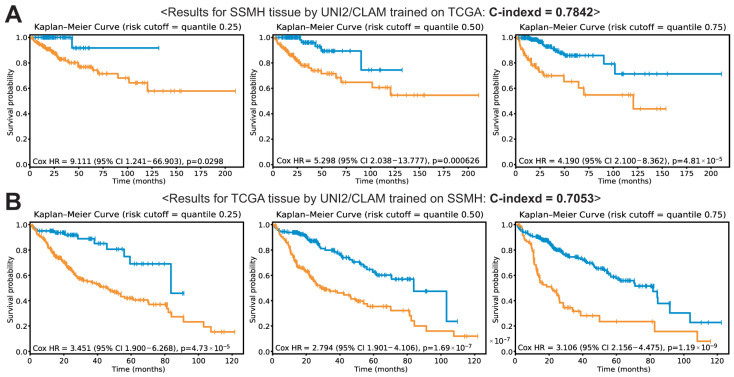
Cross-validation results for the SSMH and TCGA datasets using UNI2/CLAM trained on all tissue patches. Kaplan–Meier (KM) curves are shown for risk groups stratified by the 25th, 50th, and 75th percentiles of the model-predicted risk scores. (**A**) KM curves for the SSMH dataset using a model trained on the TCGA dataset. (**B**) KM curves for the TCGA dataset using a model trained on the SSMH dataset.

It is important to determine whether a model trained on one dataset can generalize well to other datasets. Therefore, we tested the prognostic performance of the models trained on all tissues from either the SSMH or TCGA dataset with the UNI2/CLAM framework on the other dataset. The C-indices were 0.7842 for the SSMH dataset and 0.7053 for the TCGA dataset when using models trained on the TCGA and SSMH datasets, respectively (Figure 5A,B). Notably, these performances were significantly lower than those achieved when the models were applied to their respective datasets (*p* < 0.05). These results indicate that the generalizability of the models is limited when they are applied to other datasets.

**Figure 6:**
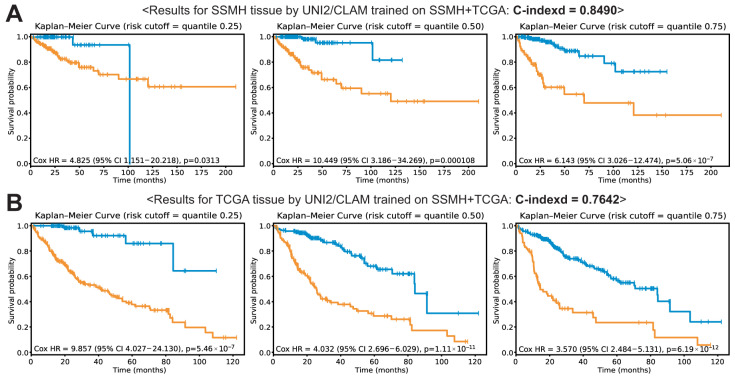
Results for the SSMH and TCGA datasets using UNI2/CLAM trained on all tissue patches from the combined SSMH and TCGA datasets. Kaplan–Meier (KM) curves are shown for risk groups stratified by the 25th, 50th, and 75th percentiles of the model-predicted risk scores. (**A**) KM curves for the SSMH dataset. (**B**) KM curves for the TCGA dataset.

Another important question is whether increasing the dataset size can improve the prognostic performance of DL models. To address this, we combined the SSMH and TCGA datasets to train a UNI2/CLAM model on all tissue regions. No improvement in performance was observed with the combined dataset, with C-indices of 0.8490 for the SSMH dataset and 0.7642 for the TCGA dataset (Figure 6).

The t-SNE analysis revealed that low-risk cases were enriched for histologic grades 1/2, whereas high-risk cases predominantly comprised grades 3/4 (Figure 3). Accordingly, we investigated whether the model’s predictions were primarily driven by tumor grade. To address this, KM analyses were performed separately within grade 1/2 and grade 3/4 subgroups with UNI2/CLAM models trained on all tissue regions (Supplementary Figure S1). In both subgroups, patients stratified into low- and high-risk groups based on model-predicted risk scores using the 75th percentile cutoff demonstrated clear survival separation. These findings suggest that the model captures prognostic information beyond histologic grade, providing a complementary prognostic signal.

To further assess the time-dependent discriminative performance of the models beyond the C-index, we performed time-dependent ROC analysis at 1-, 3-, and 5-year horizons (Supplementary Figure S2). ROC curves at these time points were generated for the SSMH and TCGA datasets using UNI2/CLAM models trained on all tissue regions and applied to the corresponding datasets. Although the AUC values gradually decreased from the 1- to 3- to 5-year horizons, the models retained meaningful time-dependent discriminative performance across all time points for both datasets.

## 4. Discussion

Conventionally, HCC prognostication has relied on machine learning approaches using clinicopathological and demographic parameters [26,27,28,29]. However, these approaches are inherently limited in their ability to capture the complex spatial architecture and heterogeneity of tumor tissues. In this context, DL approaches applied to histopathological images have emerged as a promising alternative, enabling the direct extraction of rich morphological features for improved prognostic modeling.

Numerous studies have explored DL-based prognostic prediction from histopathology in HCC. Early studies primarily relied on CNNs to learn prognostic features from tissue image patches. For example, Saillard et al. applied an attention mechanism to CNN-derived features for OS prediction and reported a C-index of 0.70 for the TCGA dataset [14]. Lu et al. employed three pre-trained CNN models—VGG16, InceptionV3, and ResNet50—to extract features from HCC histopathological images and predicted OS and disease-free survival (DFS) using TCGA-LIHC diagnostic slides [15]. They achieved C-indices of 0.789 and 0.744 for OS and DFS, respectively. Yamashita et al. predicted recurrence using MobileNetV2 and obtained C-indices of 0.724 and 0.683 for the TCGA dataset and their own dataset, respectively [19]. Zhang et al. used ResNet-50 to extract features from tissue images and further analyzed the micro- and macro-architectural characteristics of HCC tissues [17]. The C-indices for OS were 0.764 for the TCGA dataset and 0.713 for their own dataset. Shi et al. utilized ResNet-18 to classify tissue images into multiple categories and derived risk scores for OS based on the model outputs [18]. The C-indices were 0.731 for their own dataset and 0.713 for the TCGA dataset.

More recently, foundation models have been increasingly adopted for survival prediction across multiple cancer types [30]. For example, Jin et al. trained a pathology foundation model using diverse cancer types from the TCGA archive and predicted OS across multiple cancer types [31]. The C-indices for OS prediction ranged from 0.618 to 0.786, with a value of 0.776 for the TCGA-LIHC dataset. Hu et al. also adopted a foundation model-based approach to integrate histology with genomic data for pan-cancer prognosis prediction [32], reporting a relatively low C-index of 0.577 for the TCGA-LIHC dataset. Zhou et al. developed a multimodal foundation model that integrates pathology images, clinical reports, and genomic data for pan-cancer prognosis prediction [33]. Their model achieved C-indices ranging from 0.621 to 0.830 for OS prediction, with a value of 0.761 for the TCGA-LIHC dataset.

In this study, we directly compared a patch-level CNN approach with a foundation model-based MIL approach for OS prediction from histopathology in HCC. Additionally, we evaluated how the inclusion of diverse tissue components, as opposed to isolated tumor regions, affects prognostic model performance. Although the patch-level CNN approach did not perform well when all tissue regions were used, the foundation model-based MIL approach demonstrated stable performance regardless of whether it was trained on all tissue regions or only tumor regions. Prognostically relevant information in histopathology is often localized to specific tumor regions rather than uniformly distributed across the entire tissue section. Patch-level models implicitly assume that all patches contribute equally to the survival outcome, whereas attention-based MIL explicitly learns to identify and emphasize a subset of informative regions. In patch-level survival modeling, all patches extracted from a slide are assigned identical survival labels, despite substantial intra-slide heterogeneity and the absence of prognostic information in normal tissue regions. As a result, a large proportion of patches are associated with noisy or weakly informative labels, which can obscure prognostic signals and hinder the effective optimization of the Cox objective, particularly when all tissue regions are included. Therefore, an attention mechanism plays a critical role in extracting prognostically relevant signals from diverse tissue components in WSIs. In addition to its superior performance, attention-based MIL provides a degree of interpretability by highlighting regions that contribute most strongly to the predicted risk via attention heatmaps. This spatial localization enables pathologists to visually inspect prognostically relevant tissue patterns, potentially increasing trust and facilitating hypothesis generation.

Foundation models are generally considered more generalizable than CNN-based models, as they learn rich and transferable feature representations through large-scale pretraining. However, our foundation model-based MIL approach showed a marked decline in predictive performance when cross-validation was performed between the SSMH and TCGA datasets (Figure 5). Furthermore, performance did not improve when a model trained on the combined dataset was evaluated separately on each dataset (Figure 6). Limited generalizability may be attributed to domain shifts between datasets, including variations in staining protocols, scanner types, image resolution, and tissue preparation procedures, all of which can substantially affect the visual characteristics of histopathological images. Although stain normalization was applied in this study, these systematic differences cannot be entirely eliminated. To more rigorously assess the impact of staining variability, future studies should include stress-testing under simulated extreme stain variations. A systematic evaluation of performance under controlled stain perturbations would further strengthen the understanding of model robustness and its real-world clinical applicability. There are also other fundamental differences between the two datasets. First, the incidence of underlying comorbidities differs between them. In the SSMH dataset, approximately 60% of HCC cases were associated with hepatitis B virus (HBV) infection, whereas only 28% of patients in the TCGA dataset had HBV infection. Second, the median survival time among deceased patients was shorter in the TCGA dataset (14 months) compared to the SSMH dataset (19 months). These etiologic and prognostic differences may substantially influence the histomorphological characteristics of HCC between the two datasets. Therefore, the limited generalizability of DL models across the two datasets is likely inevitable given the fundamental differences between them.

In this context, the lack of performance gain by a model trained on the combined dataset appears to be an inevitable consequence of the substantial differences between the datasets. These findings suggest that simply increasing the size of the training dataset does not necessarily lead to improved model performance. Rather, the effectiveness of dataset integration depends critically on the degree of compatibility between datasets. When datasets share complementary characteristics, combining them may enhance model performance by enriching the diversity of training samples. In contrast, when datasets exhibit substantial differences in clinical, etiological, or histomorphological features, such integration may instead introduce conflicting signals, thereby limiting or even degrading model performance. Therefore, the impact of increasing dataset size should be interpreted in the context of dataset compatibility, highlighting the importance of considering domain alignment and data harmonization when aggregating multi-institutional data for DL-based prognostic modeling.

Some studies have treated prognostic modeling as a binary classification task to discriminate whether an event occurs or not [16,27]. Unlike classification, which assigns discrete labels, survival prediction involves modeling time-to-event outcomes in the presence of censored data, requiring the model to account for both whether and when an event occurs. This introduces additional complexity, as the learning process must capture temporal dynamics and handle incomplete follow-up information. Despite these challenges, survival prediction provides more clinically meaningful insights. Rather than providing binary or categorical outputs, it enables risk stratification over time, which can directly inform prognosis estimation, treatment planning, and patient counseling. Therefore, although it is more difficult to optimize and evaluate, survival prediction represents a more realistic and clinically actionable modeling objective, aligning more closely with real-world decision-making processes in oncology.

Reported C-indices for OS prediction on the TCGA-LIHC dataset were 0.577 [32], 0.700 [14], 0.713 [18], 0.761 [33], 0.764 [17], 0.776 [31], and 0.789 [15]. The C-index for the TCGA-LIHC dataset in the present study (0.7744) is among the highest reported. Furthermore, performance on the SSMH dataset was notably high, with a C-index of 0.8752. To the best of our knowledge, this represents one of the highest C-indices reported for HCC prognostication. Even small incremental gains in prognostic accuracy can have meaningful clinical implications in HCC. Improved risk stratification enables more precise identification of high-risk patients with poor outcomes, which may inform decisions regarding postoperative surveillance intensity, adjuvant therapy, and patient counseling. Notably, the model demonstrated prognostic value beyond histologic grade, suggesting that it captures complementary information not readily available through conventional pathological assessment.

Despite the strengths of this study, several limitations should be acknowledged. First, this study was based on retrospective data from only two datasets, which may limit the generalizability of the findings to broader clinical settings. Second, variability in staining protocols, scanner types, and tissue preparation procedures was not fully controlled, and stain normalization alone was insufficient to eliminate these differences. Third, although attention-based MIL provides a degree of interpretability, the biological significance of attention weights remains incompletely understood and requires further validation. Fourth, the sensitivity to the patch selection strategy has not been fully investigated. Although we compared differences between all-tissue and tumor-only paradigms, more detailed selection strategies—such as distinguishing between stromal and tumor parenchymal regions—may also have important implications for model performance. The application of additional classifiers to differentiate tissue components may further improve patch selection strategies in future studies. Alternatively, integrating pathomics features, such as nuclear atypia, tumor cell density, and trabecular thickness, may further improve model performance. Because attention heatmaps provide only indirect information and do not clearly reveal which features are decisive for survival prediction, incorporating pathomics features may help improve the interpretability and explainability of the model. Fifth, the MIL-based model relies on pretrained foundation model embeddings, and its performance is therefore dependent on the quality and domain relevance of the pretraining data. If the pretraining dataset does not adequately capture the diversity of histopathologic patterns in HCC, this may limit downstream performance. Methods such as pseudo-labeling and self-supervised refinement have been proposed to improve feature discrimination in foundation models, particularly in the context of medical image analysis [34]. While these approaches may improve representation quality, the present study focuses on evaluating the generalizability of pretrained embeddings without additional fine-tuning in a survival prediction setting. Future work may investigate whether such feature refinement strategies can further improve prognostic performance in histopathology.

Although the present study included two independent datasets and performed cross-dataset validation, the analysis remains limited by its retrospective design and the relatively small number of cohorts. The cross-dataset experiments, in which models trained on the SSMH cohort were tested on the TCGA cohort and vice versa, provide a practical leave-one-dataset-out assessment of generalizability. However, the reduced performance in these experiments indicates that the model is not yet sufficiently robust for direct clinical deployment. This limitation is particularly important because real-world clinical implementation would require stable performance across institutions with heterogeneous staining protocols, scanner systems, tissue processing procedures, patient populations, and etiologic backgrounds. The lack of performance improvement after combining the SSMH and TCGA datasets suggests that integrating only a small number of heterogeneous cohorts may lead to conflicting domain-specific signals, limiting the benefits of dataset aggregation. This finding underscores the importance of incorporating more diverse and representative multi-institutional datasets for training to enhance robustness and generalizability. Future studies should validate the model in larger prospective multi-institutional cohorts and incorporate strategies for stain harmonization, domain adaptation, and model calibration before clinical application.

In addition, this study focused solely on histopathological images for prognostic modeling. However, patient outcomes in HCC are influenced by a wide range of factors, including clinical variables, laboratory findings, radiological features, and molecular characteristics. Therefore, future studies should explore multimodal approaches that integrate histopathological images with complementary data sources to improve prognostic performance and robustness. Such multimodal prognostic modeling may enable a more comprehensive representation of tumor biology and patient status, ultimately leading to more accurate and clinically actionable predictions.

## 5. Conclusions

This study demonstrates that a foundation model-based MIL approach provides robust and interpretable prognostic modeling from histopathological images in HCC, particularly in the presence of heterogeneous tissue components. DL-based prognostic prediction can provide valuable complementary information beyond conventional clinicopathological variables, thereby enhancing risk stratification and supporting more precise clinical decision-making. Therefore, continued efforts to improve model robustness, interpretability, and generalizability, along with the integration of multimodal data, will be essential for advancing DL-based prognostic modeling toward real-world clinical application.

## Data Availability

The TCGA data presented in this study are openly available in the GDC data portal (https://portal.gdc.cancer.gov/, accessed on 15 October 2025). Further information is available from the corresponding authors upon request.

## Supplementary Materials

The following supporting information can be downloaded at https://www.mdpi.com/article/10.3390/cancers18101534/s1. Figure S1: Kaplan–Meier (KM) analyses stratified by tumor grade (grade 1/2 vs. grade 3/4); Figure S2: Time-dependent receiver operating characteristic (ROC) analysis at 1-, 3-, and 5-year horizons; Table S1: Comparison of different models and methods for survival prediction in the SSMH dataset; Table S2: Detailed information on model training and hyperparameter settings.

## Abbreviations

The following abbreviations are used in this manuscript:

AUC	Area under the curve
C-index	Concordance index
CLAM	Clustering-constrained attention multiple-instance learning
CNN	Convolutional neural network
DL	Deep learning
H&E	Hematoxylin and eosin
HCC	Hepatocellular carcinoma
HR	Hazards ratio
KM	Kaplan–Meier
MIL	Multiple-instance learning
OS	Overall survival
ROC	Receiver operating characteristic
t-SNE	t-distributed stochastic neighbor embedding
WSI	Whole-slide images
