# Supplementary material for: Comparative Analysis of Deep Learning Approaches for Histopathology-Based Survival Prediction in Hepatocellular Carcinoma
Source: Cancers (Basel). 2026 May 9;18(10):1534. doi: 10.3390/cancers18101534 (PMC13204618; doi:10.3390/cancers18101534)
Supplement: Supplementary file 1 [file cancers-18-01534-s001.zip › Supplementary Tables.pdf]

Supplementary Table S1. Comparison of different models and methods for survival prediction in the SSMH dataset

**CNN model**	**Parameters**	**C-index for all-tissue**	**C-index for tumor-only**
DenseNet-121	~8.0M	0.7388	0.8231
ResNet-50	~25.6M	0.7536	0.8224
Inception v3	~23.8M	0.7498	0.8308
**MIL method**	**Parameters for current setting**	**C-index for all-tissue**	**C-index for tumor-only**
ABMIL	526K	0.8431	0.8402
CLAM	528K	0.8701	0.8752

Supplementary Table S2. Detailed information on model training and hyperparameter settings

**Component**	**Inception v3 (Patch-level CNN)**	**CLAM (UNI2 + MIL)**
**Input unit**	Image patches (360×360, 20×)	Bag of patch embeddings (per WSI)
**Feature extractor**	Inception v3 (trained end-to-end)	Pretrained UNI2 encoder (frozen)
**Aggregation**	Mean pooling (patch-level averaging)	Attention-based MIL pooling
**Optimizer**	AdamW	AdamW
**Initial learning rate**	1e-4	2e-4
**Learning rate schedule**	Step decay	Cosine annealing
**Batch size**	128 patches	4 bag (4 WSI)
**Loss function**	Cox partial likelihood	Cox partial likelihood
**Handling of censoring**	Included via Cox loss	Included via Cox loss
**Handling of class imbalance**	Not explicitly applied	Same as left
**Handling of skewed survival distribution**	No explicit rebalancing	Same as left
**Regularization**	Dropout, weight decay (1e-5)	Dropout in attention layer, weight decay
