# Supplementary material for: Comparative Analysis of Deep Learning Approaches for Histopathology-Based Survival Prediction in Hepatocellular Carcinoma
Source: Cancers (Basel). 2026 May 9;18(10):1534. doi: 10.3390/cancers18101534 (PMC13204618; doi:10.3390/cancers18101534)
Supplement: Supplementary file 1 [file cancers-18-01534-s001.zip › Supplementary Figures.pdf]

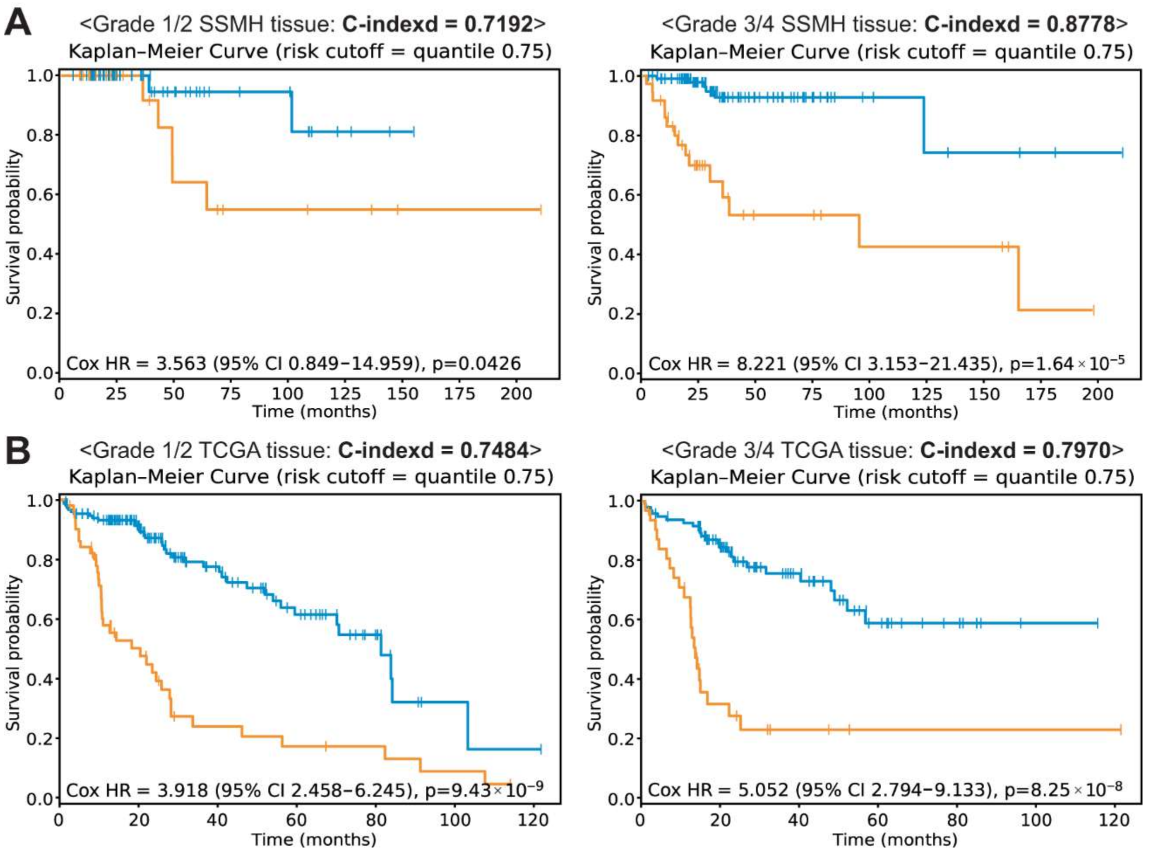

Supplementary Figure S1. Kaplan–Meier (KM) analyses stratified by tumor grade (grade 1/2 vs. grade 3/4). KM curves are shown for risk groups stratified by the 75th percentile of the model-predicted risk scores. (A) KM curves for grade 1/2 and grade 3/4 subgroups in the SSMH dataset using UNI2/CLAM trained on all tissue patches. (B) KM curves for grade 1/2 and grade 3/4 subgroups in the TCGA dataset using UNI2/CLAM trained on all tissue patches.

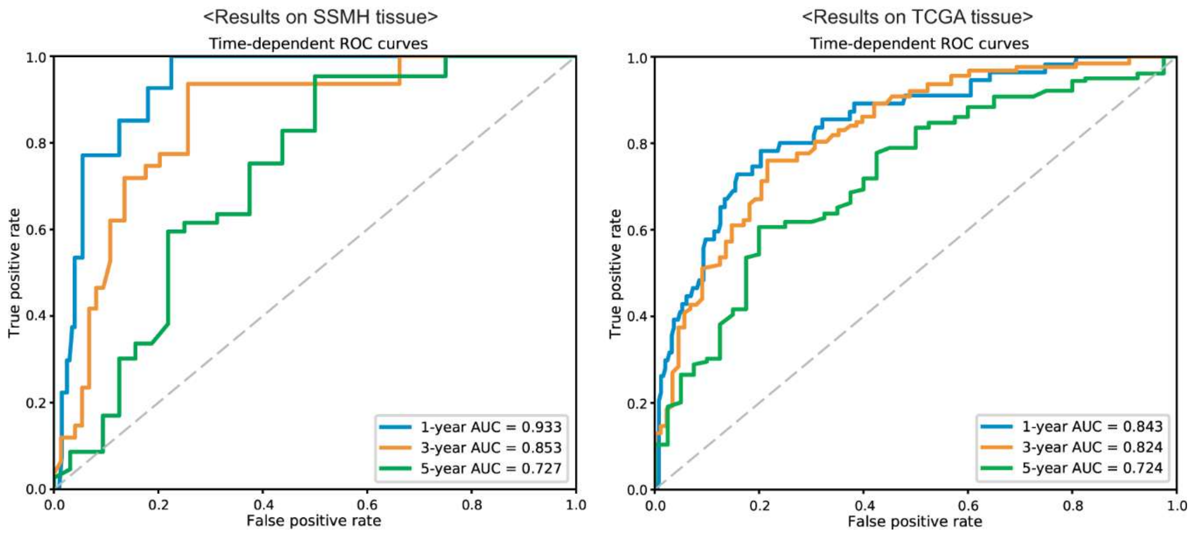

Supplementary Figure S2. Time-dependent receiver operating characteristic (ROC) analysis at 1-, 3-, and 5-year horizons. ROC curves are shown for the SSMH (left) and TCGA (right) datasets using UNI2/CLAM trained on all tissue patches.
